# Ecological Study on the Oral Health of Romanian Intellectually Challenged Athletes

**DOI:** 10.3390/healthcare10010140

**Published:** 2022-01-12

**Authors:** Arina Vinereanu, Aneta Munteanu, Alexandru Stănculescu, Alexandru Titus Farcașiu, Andreea Cristiana Didilescu

**Affiliations:** 1 Faculty of Dental Medicine, “Carol Davila” University of Medicine and Pharmacy, 032799 Bucharest, Romania; arina.vinereanu@umfcd.ro (A.V.); alexandru.stanculescu@rez.umfcd.ro (A.S.); andreea.didilescu@umfcd.ro (A.C.D.); 2 Special Olympics Romania, 077025 Bragadiru, Ilfov, Romania

**Keywords:** intellectual challenge, disability, oral health, socio-economic factors, Special Olympics

## Abstract

This study aimed to give an image of the oral health status and treatment needs of intellectually challenged athletes taking part in Special Olympics—Special Smiles (SO-SS) events organized in Romania during 2011–2019 and to find potential associations with socio-economic factors. An observational ecological retrospective study was conducted, and 1860 oral examinations were performed on participants aged 8 to 30 years in nine SS-SO events. Oral examination was performed under field conditions. Percentage of caries-free subjects, mean DMF-T index and its components (D = decayed; M = missing; F = filled; T = teeth), restoration index RI = [F/(F + D) × 100]%, and Plaque Index were calculated for each of the nine groups. Caries-free subjects ranged between 5.90% and 21.70%. DMF-T ranged from 4.27 to 7.71. Higher values for “F” component (range 0.66–1.69) and RI (range 13.02–27.74%) were found in events held in cities from regions with higher reported Gross Domestic Product. Sealants were present in 0 to 8.4% of the subjects. In areas with lower numbers of inhabitants per dentist, more sealants were found (*p* < 0.001). Romanian SO athletes exhibited relatively poor oral health, limited access to dental treatment, and low level of prevention. Targeted prevention and treatment programs adjusted to specific conditions in each geo-economic region are needed.

## 1. Introduction

Intellectual disability (ID) is characterized by significant limitations in both intellectual functioning and adaptive behavior, which covers many everyday social and practical skills, usually diagnosed before the age of 22 [1].

People with ID are reportedly more vulnerable to oral health issues [2]. Mentally challenged persons tend to have poorer oral hygiene, more periodontal diseases, higher rates of untreated caries, and more extracted or compromised first permanent molars [3] and more unmet oral treatment needs [4,5]. As sport is associated with increased masticatory muscle activity [6], this can lead to bruxism, tooth wear, and tooth sensitivity, which makes athletes with ID more prone to temporo-mandibular disorders [7].

There are many medical conditions that involve various degrees of intellectual disability. Either by own characteristics or by specific medication, some of these conditions may make their carriers more prone to dental and/or periodontal disease. For example, oral breathing (common for a large variety of intellectual disabilities [8,9]) comes with orthodontic problems and poor periodontal health, generating favorable circumstances for the development of both decay and gingival inflammation. On the other hand, gingival enlargement and subsequent inflammation that may occur as a side effect of some antiepileptic drugs (e.g., phenytoin and sodium valproate) [10] can favor gum disease and food retention. Sugary vehicles used for children’s medication, lesser abilities of patients to understand oral hygiene and to perform self-brushing, as well as parents’ limited concern regarding oral health in an already complex time- and energy-consuming overall context may count as additional factors for a more challenged oral health in children with ID [5]. All of the above point to the fact that prevention of oral disease is crucial for this category of population.

Romania currently has no national governmental programs for the prevention and/or dental treatment for children/people with ID. Special Smiles is a worldwide non-governmental oral health program developed by Special Olympics International Foundation as part of the “Healthy Athletes” initiative, which aims to improve the general health of athletes with ID involved in the Special Olympics movement. Special Olympics-Special Smiles (SO-SS) was first introduced in Romania in 2005. The program initially consisted of oral screenings combined with adapted oral hygiene training sessions for intellectually challenged athletes taking part in Special Olympics (SO) sports competitions. Since 2018, Special Smiles Romania also provides on-site professional brushing and glass-ionomer sealants.

During the last 20 years, many studies regarding the oral health status of SO athletes from different parts of the world were published [2,4,5,11,12,13,14,15,16,17]. Several papers on the oral health status of Romanian SO athletes are available, showing the trends, over time, of the oral health of this category [2,18,19].

The oral health status is reportedly influenced by various factors, such as the socio-economic status or parents’ level of education [20]. To the author’s knowledge, no studies linking the oral health of people with ID to socio-economics parameters from various regions of the country have been performed so far in Romania.

Various geographical areas of Romania have different historic backgrounds and different socio-economic levels of development [21]. These differences could influence other parameters, such as the general health of the population, including the oral health of people with ID. On the other hand, as SO-SS events generate, over time, a relatively large database on the oral health status of intellectually challenged athletes, the recorded findings could be used as indicators for the evolution of the oral health of this category of population. Previous studies suggested associations between gross domestic product (GDP) and oral health status [22,23]. The results of the screenings and potential correlations with various factors may be useful for the improvement of national and regional policies regarding oral care for people with special healthcare needs.

Given the above, the aim of our study was to give an overall image of the oral health status and treatment needs of mentally challenged athletes taking part in SS-SO national events organized in different locations in Romania and to identify potential factors that may have influenced the situation.

## 2. Materials and Methods

**Figure 1:**
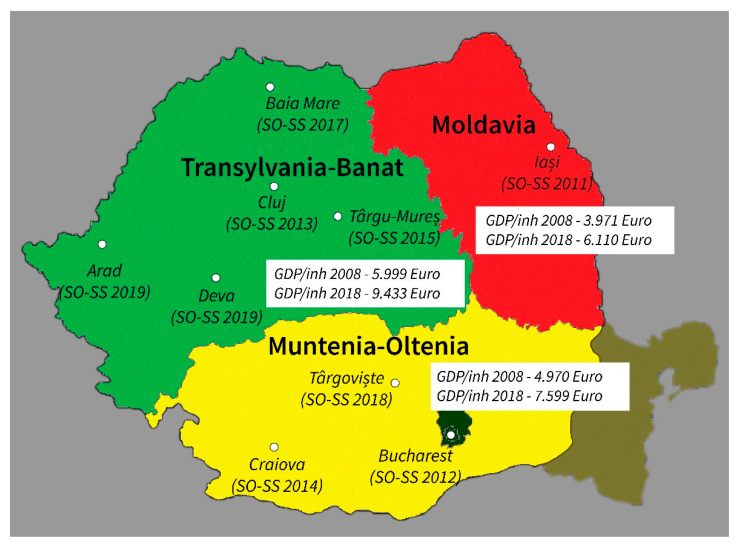
Distribution of cities hosting SO-SS events within the geo-economic regions of the country and reported GDP values for each region in 2008 and 2018 respectively.

An observational ecological study was performed using data collected from nine SO-SS annual events held in Romania between 2011–2019, in cities from different geo-economic regions of the country: Muntenia-Oltenia (Bucharest, Craiova, Târgoviște), Moldavia (Iași), and Transylvania-Banat (Cluj, Târgu-Mureș, Arad, Baia Mare, Deva) (Figure 1).

The nine respective study groups consisted of self-selected Special Olympics Romanian (SOR) athletes taking part in the Special Smiles initiative. It is important to mention that, due to logistic reasons, participants in each event mainly come from the area surrounding the host town/city; therefore, the collected data can be regarded as relevant for the respective regions, although the home region of the athletes was not individually registered. Informed consent was obtained prior to the event from the athletes’ parents or guardians, as required by the standard SO-SS protocol. Athletes participating in SO events had a minimum age of 8 years (with no maximum age limit). The only inclusion criteria for participation in SO-SS events was subjects’ own acceptance to be examined. Thus, a convenience study group resulted. In order to have a more age-compact study population and to make comparisons with previous reports more relevant, for this study, we narrowed the age range and selected from all the examined athletes only those aged between 8 and 30 years [4].

The study was approved by the Bioethics Committee of Carol Davila University of Medicine and Pharmacy from Bucharest (Protocol No. 110/07.02.2017).

Oral examinations were performed by trained volunteer dentists under field conditions, using disposable dental mirrors and probes, following the SO-SS standardized screening protocol [15]. For more detailed individual information, the mentioned SO-SS screening was doubled by a regular dental examination performed by the same examiner, under the same circumstances [24]. Examiners were either pediatric dentists or general practitioners open to working with children/people with disabilities. All volunteers had undergone a special training session the day before the event. A number of 15 to 20 volunteer dentists participated in every SO-SS event.

Demographic data (age, sex), status of each tooth (sound, sealed, decayed, restored, or extracted) were recorded for each subject. Percentage of caries-free subjects, mean values for DMF-T (decayed, missing, and filled teeth) index and its components (D = decayed teeth, M = missing teeth due to caries, F = filled teeth) and restoration index RI = [F/(F + D) × 100]% were calculated for each group [25].

Signs of gingival conditions were also individually recorded. According to SO-SS standard screening rules [26], congestion or significant deviation from the normal contour or texture of free or attached gingival margins or papillae on three or more teeth within the same area (between the lower canines) was recorded as sign of gingival disease. The plaque index (PI) [27] was calculated starting from 2012.

Data distributions were expressed as means and percentages, as appropriate. The results were tested for possible correlations with data regarding the number of inhabitants per active dentist, taken from official reports (Table 1), using Pearson correlation coefficient. Data were analyzed using SPSS 20.0. The level of statistical significance was set at *p* < 0.05.

## 3. Results

A total of 2221 oral examinations were performed during the nine SO-SS events, of which 1860 were included in the study after eligibility assessment. The overall picture of the calculated parameters for each of the nine events is given in Table 2.

The proportion of caries-free athletes varied between 5.9% (Târgoviște, 2018) and 21.7% (Craiova, 2014), while the mean DMF-T values ranged between 4.27 (Craiova, 2014) and 7.71 (Baia Mare, 2017). The “decayed” component (D) of DMF-T score varied between 3.09 and 4.88, and the “filled” component (F) ranged between 1.16 and 1.69 (except for the detached 0.66 recorded in Craiova, 2014). The “missing” component (M) varied from 0.5 to 1.67.

Presence of sealants was influenced by the reported number of inhabitants per dentist, with more sealants found by examinations carried out in cities with fewer inhabitants per dentist (*r* = −0.23, *p* < 0.001). No significant correlations were found between the reported number of inhabitants per dentist and either the restoration index (*p* = 0.450) or the mean DMF-T (*p* = 0.11).

Table 2 gives the proportion of subjects with sealants present at the time of the examination. It needs to be mentioned that for the SO-SS events in 2018 and 2019, the percentage of subjects with sealants substantially increased during the event itself, from 5% to 11% and from 4.9% to 15.2% respectively, as professional cleanings and glass-ionomer sealants are provided during SS events since 2018.

Regarding the periodontal status, the presence of clinically visible gingivitis was significantly correlated with higher values of PI (*r* = 0.54, *p* < 0.001).

The highest RI scores were found in SO-SS events organized in Cluj, Târgu-Mureș, Arad, Baia Mare, Deva—all situated in Transylvania-Banat, the region with the highest GDP reported in Romania (Figure 1).

## 4. Discussion

Among Romanian SO athletes, the proportion of caries-free subjects varied largely, from 5.90% to 21.70%. The actual caries prevalence might be even higher if more detection means (e.g., X-rays) were used. Caries prevalence indexes in our study were higher than those reported in many other similar studies. In a study conducted by Bissar et al. [28] on a sample of 160 German SO athletes (mean age = 15.3 years), the caries prevalence index was 58.1%. Fernandez et al. [12] found that 33.4% of 503 SO athletes from 53 countries of Europe and Eurasia who participated in the Special Olympics European Games held in Antwerp on October 2014 (mean age = 17.08 ± 2.2 years) had untreated caries. Trihandini et al. [4] reported untreated decay in more than 70% of a group of 1217 Indonesian SO athletes (mean age = 13.46 ± 2.97 years). In South European countries, the percentage of SO athletes with untreated caries varied between 38.1% (Spain), 46.5% (Greece), and 48.5% (Italy) [14].

The high proportion of decay among ID people can be partly explained by the fact that an individual’s cognitive and motor skills can impact the perception of need for oral hygiene and oral hygiene habits. The level of intellectual disability can limit the outcomes of self-performed oral hygiene; assistance and supervision of a parent or caregiver is almost always necessary [5] and may remain so throughout life. In this respect, caregivers’ training is an important aspect to be taken into consideration when designing oral health programs for intellectually challenged people.

Another important factor favoring caries and soft tissue diseases in people with ID can be represented by medication. Chronic administration of medicines such as psychotropic drugs may result in significant decrease in the salivary flow rate, favoring both decay and periodontal conditions [14].

In the present study, low values for F and M components of DMF-T state little previous interaction of subjects with dental professionals. Anders and Davis [3] pointed out that the levels of untreated dental decay are consistently higher in subjects with ID, several studies showing more missing and decayed teeth, but fewer filled teeth in this category than in the general population. Oliveira et al. [29] demonstrated that individuals with mental impairment not only had poorer oral health status, but also had more difficulties in getting access to oral health services than people without ID. Access to dental services for people with ID is limited by several factors, among which the high cost of dental treatment in private practice playing an important role. In countries where such services are supported by the government, access remains difficult due to long waiting lists for accessing public dental services [30].

Dental anxiety was also identified as a major barrier to accessing dental treatment for people with ID [31]. Mentally challenged individuals tend to be less cooperative than the regular patient and dentists experience more difficulty in managing them during treatment sessions [32]. Friendly encounters with dental professionals during SO-SS events may contribute to enhance the dental compliance of SO athletes.

RI gives an image of the level to which treatment needs of an individual or of a group are actually met. In the present study, mean RI values were low. Highest values of RI were found in athletes examined during the SO-SS events held in cities of Transylvania-Banat, while for Oltenia and Moldavia, RI values were much lower. Recent news regarding the national revenue per capita in 2018 mentions these latter areas of Romania among the poorest in Europe: Iași (RI 15.70%) is situated in Moldavia, in the north-eastern part of the country, a region ranking fifth among Europe’s regions with the lowest income per inhabitant (22% of the mean European income), while Craiova (RI 13.02%) belongs to the geo-historic region of Oltenia, ranking 10th within the same classification, with an income of 26% of the mean European income per inhabitant, as reported by Eurostat [21].

In contrast with the above, scarce presence of sealants in all events suggests a low preoccupation for prevention. However, the presence of sealants was better in events held in cities with fewer inhabitants per dentist. This may be regarded as sign of better access to dental care in and around these cities.

Regarding the overall low percentage of subjects with sealants on permanent teeth, it is important to note that since 2018, the SO-SS program in Romania includes professional cleaning and provision of glass-ionomer sealant, which considerably increased the proportion of subjects with sealants during the last two SS events. Data reported in other studies on SO athletes of similar ages also showed that the presence of sealants among athletes with ID was the least frequent of the oral findings [5,12,14,15,16]. The low prevalence of fissure sealants points out a persistent need for prevention. A better situation from this point of view was found by Pradhan et al. [33] in Australian SO athletes, 23% of which had sealants.

The presence of gingivitis was a rather common finding in the present study (20.8% to 79.9%). This is consistent with previous similar studies that reported signs of gingivitis in 38.7% of SO athletes from Europe and Eurasia (mean age = 17.08 ± 2.2 years) [12], in 27.80% of American SO athletes with mean age 17.4 years [15], in 29.8% of Indonesian SO participants (mean age = 13.46 ± 2.97 years) [4], and in 59% of Australian SO participants (mean age = 15 years) [33].

The high prevalence of periodontal conditions among intellectually challenged individuals might be due to an inability to perform adequate personal oral hygiene, which leads to high levels of plaque, gingival inflammation, and periodontal disease [11]. Poor lip closure, frequently found in people with ID, may also impact the level of oral hygiene, while being an additional risk factor for both orthodontic disturbances and periodontal conditions [34].

The percentage of athletes with at least one permanent molar extracted varied between 14.60% and 35.50%. These values are consistent with those reported in studies conducted on similar groups of subjects with ID. Fernandez et al. [12] found 25.2% of subjects had missing permanent teeth due to extractions in a group of 503 SO athletes from Europe/Eurasia, with a mean age 17.08 ± 2.2 years. Another study, conducted on SO athletes in southern Europe (age 18-25 years), reported higher proportions of subjects with missing teeth due to caries: 39.8% (Spain), 40.9% (Greece), and 53.4% (Italy) [14]. Pradhan et al. [33] found that 39% of Australian SO athletes aged 8 to 18 years (mean age = 15 years) had extracted teeth.

Fernandez Rojas et al. [14] estimated that a rather high rate of extractions coexisting with low prevalence of untreated caries and filled teeth could be a consequence of the lack of coverage for oral care in public health services, where extractions would be the treatment of choice for dental caries.

The findings of the present study suggest that dental addressability of people with ID can be influenced by socio-economic factors, such as the actual number of inhabitants per active dentist in the area or the economic status of the region. Romania ranks 6th in the World Health Organization (WHO) classification of European Union (EU) countries with highest rates of unmet dental care needs, after Latvia, Portugal, Greece, Iceland, and Estonia, high above the EU-reported average [35].

SO athletes are a category that must not be regarded as representative for the entire population with intellectual disabilities living in a region or country. Practicing sports makes SO athletes more likely to be not only from the younger segment of the ID population, but also healthier, with less severe disabilities and better integrated in society than other categories of intellectually challenged individuals [15]. During SO-SS events, SO athletes benefit from oral screenings, professional prevention means, and sessions of adapted education for home oral care, all of which can contribute to increased awareness among athletes, parents, and caregivers with regard to the importance of oral health within the greater picture of general health, favoring earlier detection and limitation of oral disease. For some of the athletes, SO-SS events sometimes facilitate a first encounter with dental professionals. Under these circumstances, SO athletes can be seen as a privileged sub-group within the intellectually challenged population. Thus, it becomes obvious that, when looking at people with ID as a whole, an even worse oral health situation is to be expected than that reported by SO-SS programs worldwide [2].

The globally used standardized SO-SS protocol enables comparisons between studies performed with the same methodology in various countries and regions of the world, as the protocol is widely accepted and referred to in literature [17]. However, since problems such as bruxism or temporo-mandibular disorders have been found to be common in SO athletes [7], the standard protocol could be upgraded as to include more features, and therefore, become more detailed and individually relevant. Introducing an individual tracking system for SO athletes could substantially reduce the risk of bias and enable more reliable data comparisons.

Study limits derive mainly from the ecological design, and care should be taken when extrapolating from area to individual levels. Although all the presented SO-SS events were carried out following the same mentioned protocol, sampling bias was present. Reporting was biased by the fact that some of the athletes may have participated in more than one event, as individual tracking of subjects is not possible within the validated work-frame. Along with the lack of rigorous data regarding the exact percentage of subjects coming from the city/region where the events were organized, this shows that the results should be interpreted with care. Despite the mentioned limitations, the present study gives an image of the oral health of Romanian SO athletes, as well as the met/unmet treatment needs ratio in the various geo-economic regions of the country.

Many reports state that people with ID have poorer oral health as compared to the general population, more caries and tooth extractions, fewer fillings, greater gingival inflammation, higher rates of edentulism, less prevention, more dental anxiety, and poorer access to services [30,36]. It is known that poor oral health can cause pain, difficulty in eating, sleep disturbances, may impact social integration, and decrease self-esteem, all of which can influence an individual’s quality of life [3].

Decay and gingival disease are preventable conditions. In people with special needs, dental compliance can be limited and is likely to further decrease with the increase of treatment complexity, which makes prevention crucial. Targeted programs for primary prevention and education on oral hygiene techniques are needed, as well as dedicated facilities and trained staff for both preventive and restorative care.

## 5. Conclusions

Romanian SO athletes have many unmet dental treatment needs as compared to SO athletes from other countries, and preventive care is scarce.

There are differences in meeting SO athletes’ needs for oral treatment between various area in the country, with better socio-economic situations reflected in better oral health.

Targeted regional and national prevention programs are highly needed in Romania, with more dental practitioners trained in Oral Special Care and changes in health policies in order to improve the dental addressability of people with intellectual disabilities.

## Figures and Tables

**Table 1:** Number of inhabitants per active dentist in the respective cities of the SO-SS events as reported by the Romanian National Centre for Statistics and Informatics in Public Health in 2016 for 2014.

Region/Event	No of Inhabitants/Dentist
Iași/2011	874
Bucharest/2012	697
Cluj/2013	709
Craiova/2014	1242
Târgu Mureș/2015	1258
Arad/2016	1071
Baia Mare/2017	1374
Târgoviște/2018	2518
Deva/2019	1591

**Table 2:** Descriptive data analysis regarding the oral health status of Romanian SO athletes.

City, Year	Iași, 2011	Bucharest, 2012	Cluj, 2013	Craiova, 2014	Târgu- Mureș, 2015	Arad, 2016	Baia Mare, 2017	Târgoviște, 2018	Deva, 2019
(n)	231	92	190	226	214	205	239	219	244
Mean age (years)	18.52	17.01	17.61	15.89	17.77	18.51	18.56	16.71	17.31
Caries-free (%)	19.48	15.20	9.50	21.70	11.20	10.20	7.90	5.90	9.40
Mean DMF-T [SD]	6.64 [6.36]	6.36 [5.67]	7.65 [5.48]	4.27 [4.64]	6.09 [5.43]	6.88 [6.21]	7.71 [5.85]	7.56 [5.15]	7.08 [5.51]
Mean D-T [SD]	3.82 [4.25]	4.57 [4.64]	4.95 [4.10]	3.09 [3.49]	3.45 [3.56]	4.12 [4.16]	4.60 [4.52]	5.61 [4.43]	4.88 [4.54]
Mean M-T [SD]	1.67 [2.88]	0.53 [1.52]	1.60 [3.30]	0.50 [1.40]	1.30 [2.80]	1.10 [2.56]	1.42 [2.72]	0.94 [2.50]	0.84 [2.17]
Mean F-T [SD]	1.16 [3.28]	1.15 [2.53]	1.29 [2.12]	0.66 [1.81]	1.33 [2.37]	1.63 [3.29]	1.69 [2.74]	1.10 [2.28]	1.35 [2.95]
Mean RI (%) [SD]	15.70 [30.49]	16.68 [33.18]	20.76 [30.50]	13.02 [28.86]	25.93 [35.85]	22.68 [35.95]	27.74 [37.67]	16.03 [29.73]	18.32 [32.52]
Sealants (%)	4.30	5.40	6.30	0.00	8.40	4.90	3.80	5	4.9
Mean PI [SD]	-	1.40 [0.72]	1.52 [0.65]	0.93 [0.80]	0.93 [0.53]	1.20 [0.73]	1.30 [0.69]	1.78 [0.88]	1.49 [0.90]
Gingivitis (%)	20.8	75	81.2	41.6	56.8	70.7	74.1	79.9	66
% Subjects with at least 1 molar extracted	35.50	16.30	29.50	14.60	28.50	27.80	32.20	20.10	22.10

DMF-T: decayed, missing, filled teeth; D-T: decayed teeth; M-T: missing teeth due to caries; F-T: filled teeth, RI: restoration index; PI: plaque index; SD: standard deviation; -: not available.

## Data Availability

The data presented in this study are available from the corresponding authors upon reasonable request.
